# An Effective Method for Substance Detection Using the Broad Spectrum THz Signal: A “Terahertz Nose”

**DOI:** 10.3390/s150612103

**Published:** 2015-05-25

**Authors:** Vyacheslav A. Trofimov, Svetlana A. Varentsova

**Affiliations:** Faculty of Computational Mathematics and Cybernetics, Lomonosov Moscow State University, Leninskiye Gory, Moscow 119992, Russia; E-Mail: svarentsova@gmail.com

**Keywords:** transmitted and reflected THz pulse with a few cycles, pulsed Time-Domain Spectroscopy, spectral dynamics analysis method, detection and identification of explosives, integral correlation criteria

## Abstract

We propose an effective method for the detection and identification of dangerous substances by using the broadband THz pulse. This pulse excites, for example, many vibrational or rotational energy levels of molecules simultaneously. By analyzing the time-dependent spectrum of the THz pulse transmitted through or reflected from a substance, we follow the average response spectrum dynamics. Comparing the absorption and emission spectrum dynamics of a substance under analysis with the corresponding data for a standard substance, one can detect and identify the substance under real conditions taking into account the influence of packing material, water vapor and substance surface. For quality assessment of the standard substance detection in the signal under analysis, we propose time-dependent integral correlation criteria. Restrictions of usually used detection and identification methods, based on a comparison between the absorption frequencies of a substance under analysis and a standard substance, are demonstrated using a physical experiment with paper napkins.

## 1. Introduction

Nowadays, the detection and identification of explosives, drugs and other hazardous chemical and biological agents is an urgent problem. As it is well-known, one of the more promising ways for solving this problem is by the use of terahertz radiation [1,2,3,4,5]. This is based on the fact that the frequencies of the vibrational or rotational inter-level energy transitions of many chemical compounds, including explosives, lie in the terahertz frequency range. For this reason, many hazardous materials possess unique spectral “fingerprints” in this frequency range that may be detected.

In comparison with other types of spectroscopy, THz radiation has several obvious advantages: unlike X-rays, it is non-ionizing and therefore safe for biological tissues. Many packing materials are transparent to THz radiation (plastic, paper, cardboard, cotton, *etc.*), so one can detect the substance, hidden under an opaque cover or under the clothes on the human body. Another advantage is that THz measurements are non-contact and non-invasive.

Currently, the standard method of terahertz spectroscopy is Time-Domain Spectroscopy (TDS) [6,7,8,9,10,11,12]. The method is based on the analysis of the spectrum for the terahertz signal transmitted through the substance or reflected from it. The obtained spectral characteristics of this substance (typically, these are absorption frequencies) are compared with the known absorption characteristics of a standard sample from a database. The detection and identification is based on this comparison. However, this method has obvious disadvantages. For example, many explosives have simulants—ordinary substances with a similar set of characteristic absorption frequencies—which makes the TDS method not effective at all. Opaque packaging, inhomogeneity of the substance surface, and air humidity can also make the use of this method ineffective [13,14,15].

In the present paper, we discuss a method for the detection and identification of PWM C4 plastic explosives with an inhomogeneous surface (as an example of substance under consideration) by analyzing the reflected THz signal with a few cycles. The proposed method is a continuation and development of the TDS technique; it is based on the analysis of the spectral intensities dynamics (or spectral dynamics analysis, SDA-method) at the chosen frequencies [16,17]. We emphasize that the proposed method is based on processing of the signal, reflected from or transmitted through the substance, and measured using existing THz-TDS systems.

It should be stressed that the SDA-method was successfully applied earlier in the transmission mode for the identification of explosives, including those hidden under opaque covers; substances in compound media and mixtures of substances with similar Fourier spectra in GHz and THz ranges of frequencies [18,19,20,21]. In [22,23,24,25,26,27,28,29] we showed the possibility of applying the SDA-method for the detection and identification of substances using the THz signal in reflection mode. In [26] the integral criteria of probability assessment for the detection and identification of substances were proposed. In [28,29] these criteria were applied for the detection of PWM C4 explosives with a complicated surface and the identification of two mixtures of explosives with close spectral properties in reflection mode. In [30] we showed the possibility of identifying the drugs MA and MDA with similar spectral properties, by means of the proposed modified integral criteria. In [27,31] we investigated the influence of the absolute phase of the THz pulse with a few cycles on the average spectral response. In [30,31,32] the spectral properties of THz pulses reflected from the sample placed at long distances of about 3.5 m were also investigated.

It is essential to stress that the reflected THz signals measurements for plastic explosives were performed at the Military University of Technology (Warsaw, Poland); transmitted THz signals of RDX were registered at the Center for Terahertz Research (Rensselaer Polytechnic Institute, New York, NY, USA). The measurements of reflected THz signals from paper layers at long distance were done at Moscow State University (Moscow, Russia).

We note that this article provides an overview of some results that have been previously published in [32,33] as well as new original results. Therefore, the article combines the features of a review and disclosure of novel research work in this direction.

## 2. Time-Domain Spectrometry

**Figure 1:**
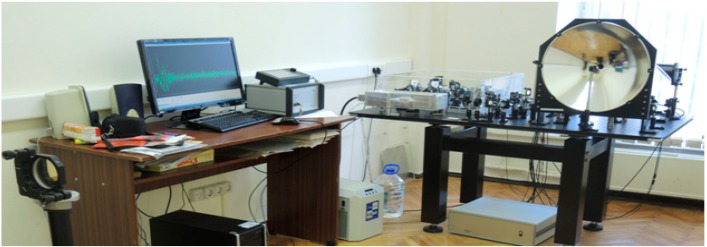
Setup for the experiment for measuring THz signals at long distance [33].

For the TDS measurement of a neutral substance (paper layers) we exploited a THz spectrometer developed by the Teravil Company (Vilnius, Lithuania). It can operate in both transmission and reflection modes. The spectral range of the spectrometer is 0.1–5.0 THz. To provide for measurements at long distance, an additional part was developed. The experimental setup is shown in Figure 1. We use a parabolic mirror focusing the THz beam on the object. Because the fiber femtosecond laser has average power of about 1 W and the laser beam is split many times, we use an additional flat mirror behind the object. Therefore, our setup operates simultaneously in reflection-transmission mode.

**Figure 2:**
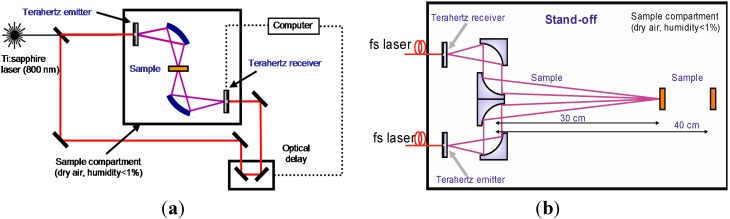
TDS transmission setup (**a**); Reflection stand-off setup (**b**).

The measurements were carried out in real conditions with a temperature 18 °C and a relative humidity of about 50%–60%. The distance between the parabolic mirror and the object was about 3.5 m. For TDS measurements of PWM C4 and other explosives (RDX, HMX, PETN), the Teraview TPS 3000 unit was used in transmission (Figure 2a) and reflection (Figure 2b) mode.

Its parameters are: spectral range 0.06–3.6 THz, signal-to-noise better than 4000:1, dynamic range higher than 3OD in the range 2 cm^−1^ to 100 cm^−1^, spectral resolution is 0.06 THz and rapid scan mode is 30 scans/s. Stand-off measurement of PWM C4 pellet response [28] was carried out in an external free standing module with a fiber-fed emitter and detector (Figure 2b). In this case, the THz beam is focused by a set of mirrors on the sample and after specular reflection is collected by the detector. In both cases of transmission and reflection mode measurements were made in the distances 30 cm between a sample (target) and the mirror.

## 3. SDA-Method and Modified Integral Correlation Criteria

### 3.1. Background of the SDA-Method

**Figure 3:**
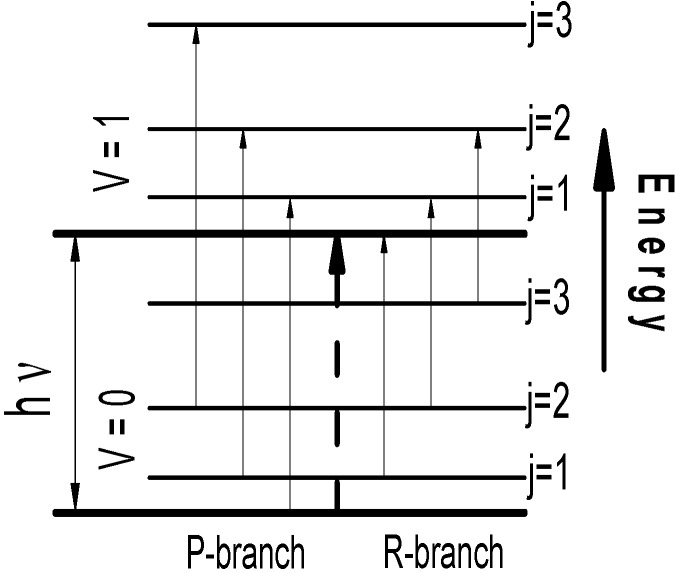
Diagram of energy inter-level transitions for a diatomic molecule.

As it is well-known, typically a broadband THz pulse with a duration of about 10 picoseconds falls on average and excites many energy levels of a molecule simultaneously. The frequencies, corresponding to transitions between these energy levels belong to the spectral range of the acting pulse: from 30 GHz up to 3–5 THz. Figure 3 shows schematically such energy level transitions for a diatomic molecule. It should be emphasized that the energy transitions with frequencies, which are greater that the spectral range of the acting pulse, are also possible due to cascading (not multi-photon) transitions from excited levels to higher energy levels. Because of this, the transmitted signal spectrum may differ significantly from the incident pulse spectrum.

Excited energy levels relax with different relaxation times through the allowed energy level transitions. Therefore, the absorption THz pulse energy may occur simultaneously with the substance emission, if the relaxation time of the corresponding energy level is less than the pulse duration. In turn, this radiation may also induce new energy transitions. Obviously, a part of the excited energy levels relaxes through indirect transitions, which leads to increased temperature of the molecule, or leads to emission with frequencies not belonging to the frequency range under consideration. As a result, a dip corresponding to this absorption frequency is formed in the spectrum. However, in the spectrum of the registered signal the radiation at this frequency may be present due to the subsequent relaxation of the higher energy levels through the energy level transition corresponding to the absorption frequency.

Therefore, one can observe absorption frequencies which are very different from the absorption spectral lines, corresponding to the spectrum of the transmitted signal registered within 10 ps (duration of the incident THz pulse) at different time intervals. Thus, the spectrum of the registered signal (reflected or transmitted) may differ significantly from that of the incident signal depending on the registered pulse duration. The proposed spectral dynamics analysis method (SDA-method) allows us to take all these features into account.

#### 3.1.1. Disadvantages of the Standard TDS Method

**Figure 4:**
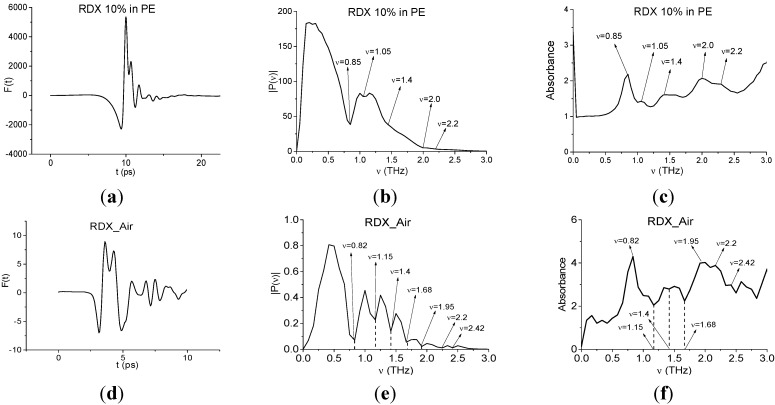
Transmitted THz signals RDX10 (**a**); RDX_Air (**d**); their Fourier spectra (**b**,**e**) and absorbance (**c**,**f**).

At present the THz TDS method, widely used for identification problem,s analyzes the spectrum of terahertz signals registered at time intervals slightly longer than the pulse duration. As an example, Figure 4 shows a THz signal transmitted through a tablet containing 10% RDX and 90% polyethylene (RDX10, for brevity): (a), its Fourier spectrum (b) and absorbance *A* (c) of a measured THz signal. Here, absorbance of a substance is defined as:

$$A=-\log_{10}(|P(\text{ν})|/|P_{REF}(\text{ν})|)$$

where |*P*(ν)|, |*P_REF_*(ν)| are the absolute values of spectral amplitudes of the measured and reference signals, correspondingly [34]. Tablet weight was 400 mg, measurements were carried out at room temperature in a chamber containing less than 2% water vapor (ideal conditions) [35].

Figure 4d shows an example of a THz signal transmitted through the tablet also containing 10% RDX and 90% PE in ambient air (RDX_Air signal). The measurements were performed at 22 °C and a relative humidity of about 50%. Note that the Fourier spectrum minima Figure 4b of the signal in the ideal conditions coincides with the maxima of the absorbance Figure 4c. This is important, because when identifying substances we use only the reflected or transmitted signal so our method is reference-free. This greatly speeds up long signal duration measurements. Usually, the absorption frequencies of the sample under investigation are compared with the absorption frequencies of standard substances, which were measured in ideal conditions. In real conditions, the THz energy absorption frequencies of water vapor may be present in the spectrum. Figure 4b shows such an example. The Fourier spectrum of the THz signal Figure 4e contains minima at frequencies ν = 1.15, 1.4, 1.68 THz. At the same time, the absorbance maxima at these frequencies are absent (Figure 4f), *i.e.*, these minima are false absorption frequencies in the Fourier spectrum of the RDX_Air signal. Their appearance is caused by the signal energy absorption by water vapor. However, the Fourier spectrum minima of the signal RDX_Air at frequencies ν = 0.82, 1.95, 2.2, 2.42 THz (Figure 4e) coincide with the absorbance maxima (Figure 4f), and they can be used for identification of the explosive RDX. Consequently, the method should be able to filter the absorption frequencies which correspond to a standard substance.

Another problem arising from the use of the standard TDS method is associated with the measurement of the THz signal in real conditions at long distances (3–6 m) in ambient air with high humidity. In Section 4.1 we present such an example of a THz signal which has passed through several layers of thin paper in ambient air with a humidity of about 50%. It is very important that the absorption spectrum of this signal has minima corresponding to the absorption frequencies of many dangerous substances—explosives and drugs. However, in our case the sample with paper layers does not contain drugs or explosives. Therefore, we claim that using the standard THz spectroscopy method for the signal analysis, measured in real conditions, leads to absurd results—one can detect hazardous substances where they are absent—for example, in a sample with paper.

#### 3.1.2. Advantages of the Spectral Dynamics Analysis Method (SDA-Method)

It is obvious that the standard THz TDS method does not take into account the instantaneous spectral intensity changes; it provides information about the spectrum averaged over the pulse registration time. At the same time, the average response to the action of the THz pulse with a few cycles is essentially non-stationary. The analysis of the spectral intensities’ evolution in time (spectral dynamics) at the chosen frequency ν allows us to get much more information about the substance than the spectrum analysis alone.

**Figure 5:**
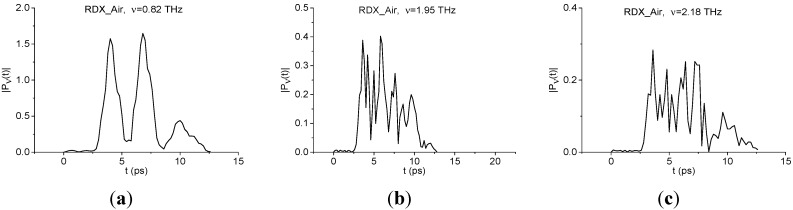
Time-dependent evolution of square root of the spectral intensity at frequencies ν = 0.82 THz (**a**); 1.95 THz (**b**); 2.18 THz (**c**) for the RDX_Air signal with a duration of about 10 ps.

Figure 5 shows the spectral lines dynamics (dynamics of square root from the spectral intensity) |*P*_ν_(*t*)| at the characteristic absorption frequencies of the explosive RDX ν = 0.82, 1.95, 2.2 THz, calculated for the RDX_Air signal with duration of about 10 ps [33]. As it can be seen, each dynamics has its own individual shape caused by both of energy absorption during the pulse action and radiation emission. The spectral dynamics contain information about not only about the spectral amplitudes’ evolution, but also information about the relaxation times of excited energy levels, if we analyze a long enough signal—about 100 ps. Thus, the spectral dynamics analysis method (SDA-method) allows us to obtain an individual 2D signature of the substance in the frequency- time domain.

Let us note that the standard THz TDS method is often sufficient for substance identification if the transmitted THz signal is obtained under ideal conditions. However, in real conditions this method is ineffective because the THz signal has a noisy Fourier spectrum, which is distorted by water vapor or by the packaging material influence, and so on. Therefore, the comparison of the corresponding frequencies between THz spectra is impossible. In this case, it becomes necessary to develop new criteria that are not based on this comparison for probability evaluation of the substance’s presence. These effective criteria can be, in particular, the integral correlation criteria [30,31,32] proposed on the basis of the SDA-method.

It is important to emphasize that even a very noisy signal contains information about the absorption frequencies and relaxation times of its energy levels, which can be determined by analyzing the spectral intensity dynamics. The first is achieved by using the spectral dynamics of the standard THz signal at its characteristic absorption frequency. These dynamics move along the spectral dynamics of the signal under investigation during the chosen time interval. As a standard signal, we use a THz signal transmitted through the substance under ideal conditions, or a signal measured in the air with water vapor. Analyzing the integral correlation between these spectral line dynamics, we can conclude about the presence or absence in the sample of corresponding absorption frequencies of the standard substance.

The resolution of the method used is a very important issue. Obviously, the frequency resolution depends on the time interval T for which the measurement is performed. As a rule, this interval is equal approximately to 100 ps. Consequently, the frequency resolution Δν of computer processing is 10 GHz. The maximum resolved frequency ν_max_ in the spectrum depends on the scanning time step Δ*t* and may be increased by decreasing Δt. However, in practice the working spectral range is limited by the spectrum generation and the spectral sensitivity of the detector, therefore Δ*t* is bounded below by the physical parameters of the equipment setup.

As it is well-known [35,36,37], over the past years methods for the detection and identification of substances on the basis of so-called electronic sensors, which are called “electronic noses” have also been developed. We recall that the “electronic nose” is a multi-sensor system for the detection and analysis of multi-component gas mixtures. Identification in the modern sensor system occurs on the basis of chemical or physical sensor properties’ changes (e.g., a change in conductivity, the change of mass, fluorescence, *etc.*). Disadvantages of this technology are well-known and are associated with the identification of hidden objects, for example, if packed in polyethylene or paper, or hidden under clothing. The implementation of the substance identification in the sensor system consists of several successive steps: data preprocessing, selection of distinctive characteristics of the substance, comparison of these characteristics with the database, decision making. The same steps are used for identifying substances using THz radiation. Therefore, by analogy with the “electronic nose”, the proposed method can be called “terahertz nose”. Its essential characteristic is the computer processing of the THz signal based on the SDA-method. It should be stressed that besides the sensor applications, the demonstrated ultrafast THz spectroscopy approach also provides new opportunities to study fundamental science in many technologically-related materials ranging from nanostructures to strongly correlated electron materials, some recent literature are [38,39].

### 3.2. The Spectral Dynamics Analysis Method (SDA-Method)

Below we review briefly the technique of obtaining the spectral intensity (or spectral amplitudes) dynamics. The modified criteria are the integral criteria, based on the analysis of total correlation characteristics over the relatively short time interval and taking into account the spectral intensity on frequencies ν_1_ and ν_2_ during this time interval. Here ν_1_ is a chosen frequency of the signal under investigation and ν_2_ is a known absorption frequency of the standard signal. The correlation characteristics in turn, are based on spectral amplitude dynamics for THz signal under investigation at the chosen frequencies.

Let *F*(*t*) be the transmitted or reflected THz signal. This signal is measured in the time interval [*t_b_*, *t_e_*], where *t_b_* and *t_e_* denote its beginning and the end, respectively. Information about of the full spectrum evaluation or evolution of its part can be obtained by sliding the time window with duration (length) *T* along the signal. At each step, the time window is shifted on the chosen time interval Δ, and then the Fourier transform is applied to the function *F*(*t*) in this window. To avoid the spectrum from “spreading”, we multiply the signal *F*(*t*) by function *g*(*t*), which tends to zero very quickly at the window edges. In order to construct the spectral line dynamics (or modulus of spectral amplitude evolution) of the function *F*(*t*) at chosen frequency *ν*, the Fourier-Gabor transform is carried out in each time window:

(1) $$P(\text{ν},t_{j})=\frac{1}{T}\int_{t_{j}}^{t_{j}+T}F(t)\cdot g(t)e^{-i2\text{πν}(t-t_{j})}dt,\;g(t)=e^{-{\left(\frac{t-t_{c}}{0.5T}\right)}^{k}}$$

where *t_j_* is a time of window beginning, *j* is a serial number of window, ν is a frequency. The units of *t_j_*, *T*, Δ and *ν* are ps and THz, respectively. Then, we calculate the spectral amplitude modulus |*P*(ν, *t_j_*)| in each time interval, for example, in the following way in order to align the beginning of the physical pulse and its representation in the SDA-method:

(2) $$\left|P_{\text{ν}}(t_{\;j})|=|P(\text{ν},\;t_{j}+T)\right|$$

Below, accordingly to our previous investigations [16,17,18,19,20,21,22,23,24,25,26,27,28,29,30,31,32,33] we choose the parameters of window duration, its shift and power *k* as: *T* = 2.8 ps, Δ = 0.2 ps and *k* = 20, respectively.

### 3.3. Modified Integral Correlation Criteria

In this section, we denote by *S*(*t*) a THz signal under analysis, and by *s*(*t*) the standard THz signal. For assessment of the integral correlation between the spectral lines dynamics for the reflected or transmitted THz signal *S*(*t*) and the standard transmitted signal *s*(*t*) at the detected frequencies, we introduce the following notations: we denote the discrete set of absolute values of spectral amplitudes for the standard transmitted signal *s*(*t*) at the chosen frequency ν (see Equations (1) and (2)) as *p*_ν_ = {|*p*_ν_(*t_m_*)|}, *m* = 1, …, *M*_1_. The corresponding set for the reflected (or transmitted) THz signal *S*(*t*) is denoted as *P*_ν_ = {|*P*_ν_(*t_m_*)|}, *m* = 1, …, *M*_2_, and its part containing *M*_1_ components, which begins at point *t_n_*, as $P_{\text{ν}}^{\left(n\right)}=\{|P_{\text{ν}}^{\left(n\right)}\;(t_{n+m})|\}$. Here *M*_1_ and *M*_2_ depend on the dynamics construction parameters—the window length T and its shift Δ, see Equations (1) and (2).

Both sets *p*_ν_ = {|*p*_ν_(*t_m_*)|} and $P_{\text{ν}}^{\left(n\right)}=\{|P_{\text{ν}}^{\left(n\right)}\;(t_{n+m})|\}$ must be averaged at each step *t_n_* to avoid their constant component influence on the correlation coefficient value. Moving the set *p*_ν1_ along the set *P*_ν2_, we get in each point *t_n_* the correlation coefficient:

(3) $$c_{p,P}(t_{n})=\sum_{m=0}^{M_{1}-1}\left(|p_{{\text{ν}}_{1}}(t_{m})|-\bar{p_{{\text{ν}}_{1}}})\cdot (|P_{{\text{ν}}_{2}}(t_{m+n})|-\bar{P_{{\text{ν}}_{2}}}\right)/||p_{{\text{ν}}_{1}}-\bar{p_{{\text{ν}}_{1}}}||\cdot ||P_{{\text{ν}}_{2}}^{\left(n\right)}-\bar{P_{{\text{ν}}_{2}}}||$$

where $\bar{p_{{\text{ν}}_{1}}}=\sum_{m=0}^{M_{1}-1}\left|p_{{\text{ν}}_{1}}(t_{m})\right|/M_{1}$, $\bar{P_{{\text{ν}}_{2}}}=\sum_{m=0}^{M_{1}-1}\left|P_{{\text{ν}}_{2}}(t_{m+n})\right|/M_{1}$. Then, using the correlation coefficient *cW_p,P_*(*t_n_*) for two spectral dynamics, we construct the integral correlation criterion [28,29]:

(4) $$C_{p,P}(t_{n})=\sum_{m=0}^{n}\left|c_{p,P}(t_{m})\right|,\;n=0,\ldots ,M_{2}-M_{1}$$

To increase the efficiency of criterion Equation (4), we develop its modification, which takes into account the spectral intensity at each of frequencies ν_1_ and ν_2_ during the interval of correlation:

(5) $$CW_{p,P}(t_{n})=\sum_{m=0}^{n}\left|c_{p,P}(t_{m})\right|w_{1}w_{2},\;n=0,\ldots ,M_{2}-M_{1}$$

where *w*_1_ = *w*(|*P*(ν_1_)|), *w*_2_ = *w*(|*P*(ν_2_)|) are the weight coefficients. This criterion is named as modified criterion.

Let us note that one can use another criterion, which deals the sets $p_{\text{ν}1}^{2}$ = {|*p*_ν1_(*t_m_*)|^2^}, $P_{\text{ν}2}^{2}$ = {|*P*_ν2_(*t_m_*)|^2^} and can be written in the following way:

(6) $$CW_{p,P}^{SQ}(t_{n})=\sum_{m=0}^{n}\left|c_{p^{2},P^{2}}(t_{m})\right|{w_{1}}^{2}{w_{2}}^{2},\;n=0,\ldots ,M_{2}-M_{1}$$

If *w*_1_ = 1, *w*_2_ = 1, we get integral characteristic Equation (4), introduced in [29].

## 4. Results and Discussion

### 4.1. Identification of Paper Layers at a Long Distance

#### 4.1.1. Spectral Properties of Paper Layers Sample

**Figure 6:**
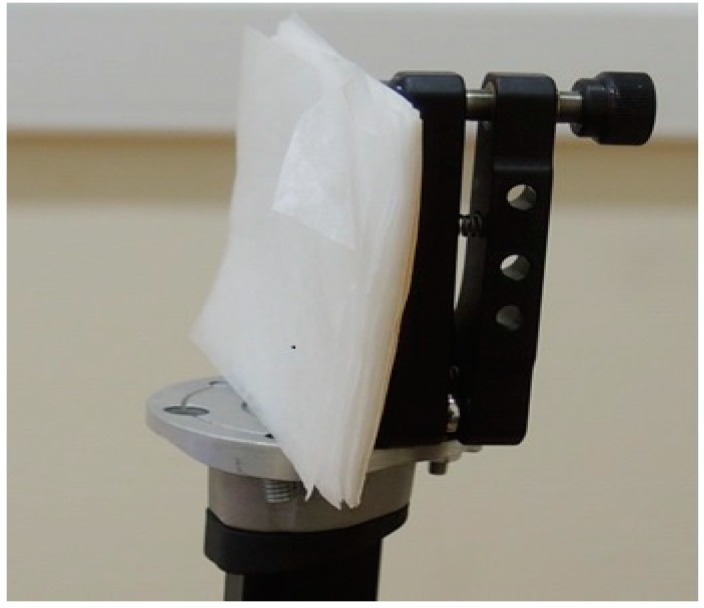
Sample with thin paper layers.

In Figure 6a we show the sample investigated under real conditions at a long distance of about 3.5 m. The sample consists of several layers of thin paper (napkins) with a total thickness of 5–7 mm. We shall call this signal the Paper Layers signal.

**Figure 7:**
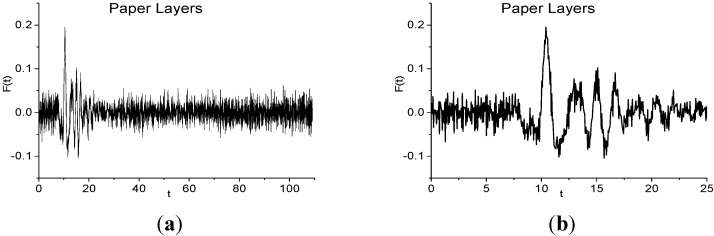
THz Paper Layers signal in the time interval *t* = [0, 110] ps (**a**); [0, 25] ps (**b**).

In Figure 7a the THz Paper Layers signal is presented for the time interval *t* = [0, 110] ps. One can see the absence of pronounced sub-pulses that are typical for reflected THz signal, and the high noise in the signal. In Figure 7b the main pulse is shown in the time interval *t* = [0, 25] ps.

**Figure 8:**
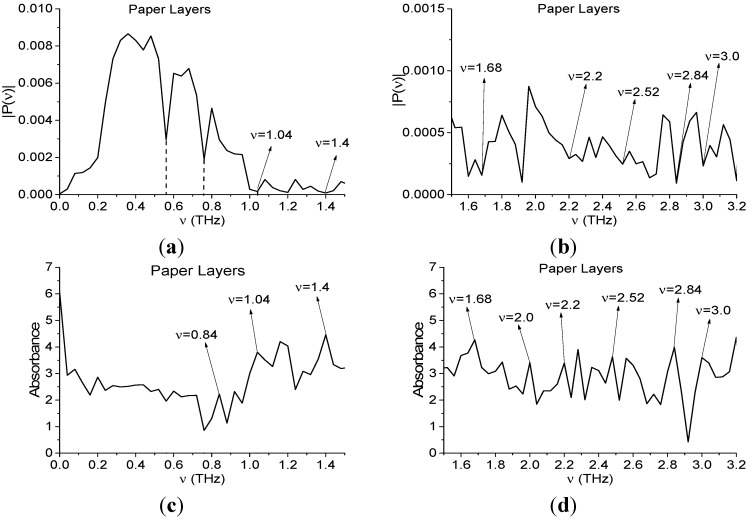
Fourier spectrum (**a**,**b**) and absorbance (**c**,**d**) of the main pulse of the signal Paper Layers in the frequency range ν = [0, 1.5] THz (a,c); [1.5, 3.2] THz (b,d).

As we have emphasized above, the standard TDS method deals with the main pulse of transmitted or reflected THz signal. Taking into account this circumstance, we show in Figure 8 the Fourier spectrum of the Paper Layers main pulse in the frequency range ν = [0, 1.2] THz (Figure 8a), [1.1, 3.0] THz (Figure 8b). In Figure 8c,d the corresponding absorbance is depicted. In [33], we showed that the spectrum minima at frequencies ν = 0.56, 0.76 THz in (Figure 8a) are caused by water vapor in the air. The absence of maxima at these frequencies in absorbance (Figure 8c) confirms our conclusion.

It is obvious that hazardous substances are absent in the paper layers. However, the Fourier spectrum and absorbance demonstrate the same spectral properties as many dangerous substances. Indeed, according to [10], the characteristic absorption frequencies of the explosives RDX, HMX, and PETN are: ν = 0.82, 1.05, 1.36, 1.54, 1.95, 2.19 THz for RDX; ν = 1.78, 2.51, 2.82 THz for HMX; ν = 2.0, 2.16, 2.84 THz for PETN.

One can see in Figure 8a,b minima and in Figure 8c,d maxima at frequencies ν = 0.84, 1.04, 2.0 THz, close to absorption frequencies of RDX; at frequencies ν = 2.52, 2.84 THz, close to those of HMX and at frequencies ν = 2.0, 2.84 THz, close to those of PETN. Let us note also that the extremes ν = 1.4, 1.68 THz in Figure 8a–d are close to characteristic absorption frequencies of the illicit drug MDA [32]. Based on these results only, it is possible to make an incorrect conclusion about the presence of explosives and drugs in the paper layers. This example shows that the TDS method, based on the spectrum analysis, is not only insufficient for the substance identification in the real conditions, but may incorrectly interpret the information obtained.

However, modified integral criteria allow us to show the absence of explosives in the paper layers. For this purpose, we will use the transmitted RDX_Air signal as the standard one. The absorption frequencies of the RDX_Air signal (see Figure 4) are in a good agreement with the frequencies given in [10]; we see the slight shift for ν = 1.05 THz ([10)] to ν = 1.15 THz (RDX_Air), frequency ν = 1.36 THz is shifted to ν = 1.4 THz, ν = 2.19 THz is shifted to ν = 2.2 THz. In Figure 4 we also observe the minimum in the Fourier spectrum and maximum of absorbance of the RDX_Air signal at frequency ν = 2.42 THz. At the same time, the Paper Layers signal spectrum in Figure 8a does not contain a minimum at a frequency which is equal or close to the main characteristic minimum of the RDX_Air spectrum at ν = 0.82 THz. Additionally the minimum at ν = 2.42 THz is also absent in this spectrum, so in order to confirm the absence of RDX in the Paper Layers sample with the help of the modified criteria Equations (5) and (6), we can use the spectral line dynamics of the RDX_Air signal at frequencies ν = 0.82, 2.42 THz as standard spectral lines dynamics.

**Figure 9:**
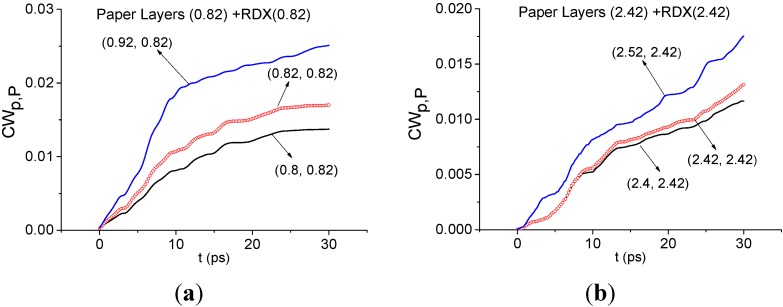
Integral characteristics *CW_p,P_*(*t_n_*) detecting the frequencies ν = 0.82 THz (**a**); 2.42 THz (**b**) THz for Paper Layers signal with RDX_Air as a standard signal.

In Figure 9 the integral criteria *CW_p,P_*(*t_n_*) evolution is shown detecting the frequencies ν = 0.82 THz (Figure 9a), 2.42 THz (Figure 9b) THz for the Paper Layers signal by using RDX_Air as a standard one. In both cases these frequencies are not detected—in each frequency range there is the criterion evolution, which lies higher than the corresponding values for the standard frequency. Therefore, we do not see a high correlation of the spectral lines dynamics of the Paper Layers and RDX_Air signals at frequencies ν = 0.82, 2.42 THz (Figure 9a,b). It means that the explosive RDX is absent in the Paper Layers sample. The result obtained for ν = 0.82 THz, is particularly important because the absorbance contains a maximum at close to the frequency ν = 0.84 THz (Figure 8c). In the same way, it is possible to show the absence of the explosives HMX, PETN and the illicit drug MDA (ecstasy) in the sample.

Here and below we assume the frequency ν is detected if the corresponding characteristic *CW_p,P_*(*t_n_*) or $CW_{p,P}^{SQ}\;(t_{n})$, calculated for the pair (ν, ν_1_) lies above all other characteristics in the frequency detection range (FDR). Here frequency ν_1_ is a chosen frequency of the standard signal. As a rule, the boundaries of the FDR are extremes of the spectrum closest to the frequency under investigation. *Vice versa*, the frequency ν is not detected if there is at least one other characteristic lying above it in this range.

It easy to show that in the time interval *t* = [25, 110] ps, which does not contain the main pulse, the spectral properties of RDX are also absent in the Paper Layers signal. We emphasize that the integral characteristics $CW_{p,P}^{SQ}\;(t_{n})$ Equation (7) give the same result as the characteristics *CW_p,P_*(*t_n_*), but the contrast of the corresponding curves increases. Their use is more preferable if the corresponding lines of *CW_p,P_*(*t_n_*) for different pairs of frequencies are close to each other ([29,30]).

#### 4.1.2. Is There Paper in the Sample with Paper Layers?

**Figure 10:**
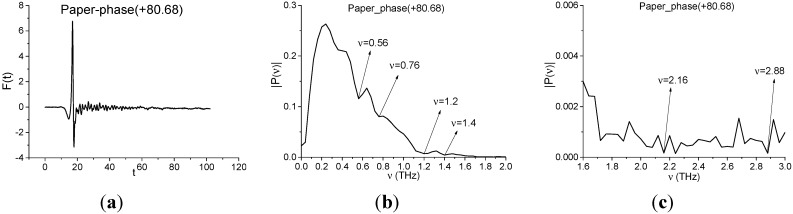
The transmitted THz Paper_phase(+80.68) signal (**a**); Fourier spectrum of the main pulse in the frequency range ν = [0, 2.0] THz (**b**); [1.6, 3.0] THz (**c**).

Below we investigate the question of paper detection in the Paper Layers signal by means of the integral correlation criteria Equations (5) and (6). For this purpose we use the standard transmitted THz signal Paper_phase(+80.68), which was measured at South China Normal University (Guangzhou, China) [27]. Figure 10 shows the transmitted THz Paper phase(+80.68) signal (Figure 10a) and the corresponding spectrum of the main pulse in the frequency range ν = [0, 2.0] THz (Figure 10b) and [1.6, 3.0] THz (Figure 10c). Comparing the Fourier spectrum of the standard signal (Figure 10) with the spectrum of the long-distance Paper Layers signal (Figure 8), one can see the common or close minima at frequencies ν = 0.56, 0.76, 2.2, 2.8 THz. To find paper features in the Paper Layers signal, we use the standard spectral lines dynamics of the transmitted Paper_phase(+80.68) signal at frequencies ν = 2.16, 2.88 THz. Despite the common minima at frequencies ν = 0.56, 0.76 THz, we do not use them (see Section 4.1.1).

**Figure 11:**
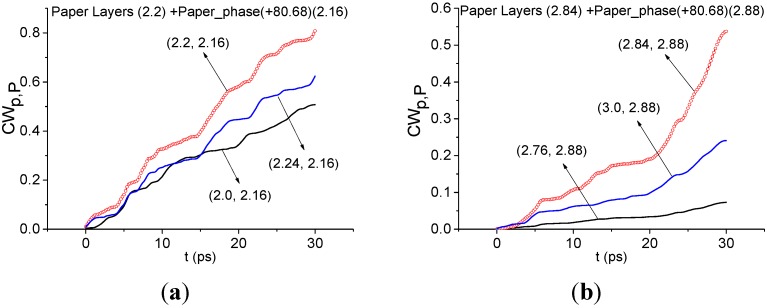
Integral characteristics *CW_p,P_*(*t_n_*) detecting the frequencies ν = 2.2 THz (**a**); 2.84 THz (**b**) for Paper Layers signal using the Paper_Phase(+80.68) signal as a standard one.

In Figure 11 the integral characteristics *CW_p,P_*(*t_n_*) are shown for the pairs ν = (2.2, 2.16) THz (Figure 11a), (2.84, 2.88) THz (Figure 11b) of signals under consideration. Both frequencies in (Figure 11a,b) are detected in the frequency ranges ν = [2.0, 2.24] THz (Figure 11a), [2.76, 3.0] THz (Figure 11b). Therefore, paper is found in the Paper Layers signal.

### 4.2. Identification of PWM C4 Explosive with Inhomogeneous Surface in Reflection Mode

In the present section, we apply the modified criteria Equations (5) and (6) for the identification of the explosive PWM C4 in pellets with inhomogeneous surfaces. PWM C4 is a mixture of RDX and plasticizer in the ratio 90%:10%. It is important, that we analyze the signal in the time intervals, which do not contain the main pulse of the reflected THz pulse. The distance between the sample and detector is 30 cm.

#### 4.2.1. Rough Surface

**Figure 12:**
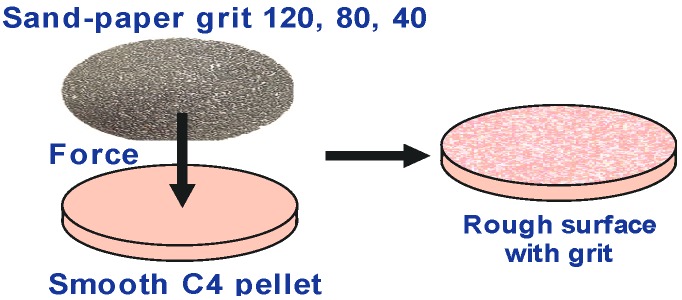
Surface treatment of the PWM C4 tablet [32].

In Figure 12 we show the PWM_C4 pellet with rough surface treated with 120, 80 and 40 grit sand paper (the root-mean squared height of the grain is 40, 60 and 130 μm, respectively). The weight of all pellets is 600 mg.

**Figure 13:**
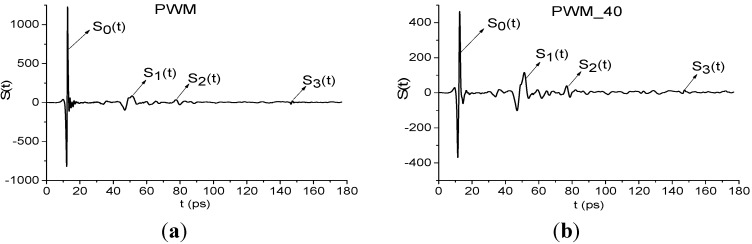
Reflected THz PWM signal (**a**) and PWM_40 signal (**b**) in the long time interval 0 < *t* < 180 ps.

All measured reflected signals (denoted as *S_R_*(*t*)), are long-term with duration about 180 ps. We shall call them the PWM_120, PWM_80, PWM_40 signals for rough C4 pellets, correspondingly, and the signal PWM for the initial smooth C4 pellet, see Figure 13a,b. The signals *S_R_*(*t*) are composed from several pulses—the main pulse *S*_0_(*t*) reflected from the outer surface of the sample, and the visible sub-pulses *S*_1_(*t*), *S*_2_(*t*), *S*_3_(*t*) due to multiple internal reflection from inner surfaces. Note that we shall consider the averaged signals:

(7) $$S(t)=S_{R}(t)-\frac{1}{T_{1}}\int_{0}^{T_{1}}S_{R}(t)dt$$

where *T*_1_ = 180 ps is the reflected signal duration. This allows us to eliminate the constant component of the signal and enhance the correlation contrast.

**Figure 14:**
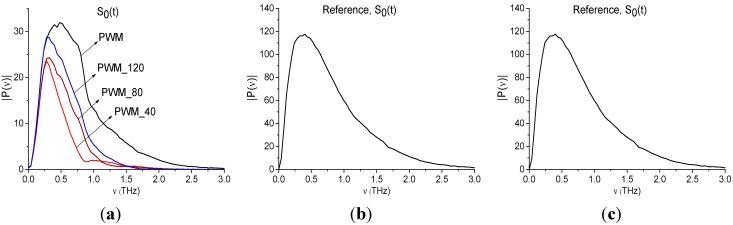
Fourier spectra (**a**) and reflectance (**b**) of the main pulses *S*_0_(*t*) of the PWM_40, PWM_80, PWM_120 and PWM signals; Fourier spectrum of Reference signal (**c**).

In Figure 14 the Fourier spectrum and reflectance of the main pulse *S*_0_(*t*) of reflected signals PWM and PWM_120, PWM_80, PWM_40 are shown. Reflectance *R* is calculated as the ratio [33]:

*R* = |*P*(ν)|/|*P_REF_*(ν)|

Here *P*(*ν*), *P_REF_*(*ν*) are spectral amplitudes at the frequency ν for the main pulse of the PWM signal and Reference signal, correspondingly. Note that the THs signal, reflected from the gold mirror was used as Reference signal.

We see that both characteristics strongly depend on the explosive shape; the pronounced identifiers of RDX are absent in both the spectra (Figure 14a) and reflectance (Figure 14b). Therefore, an inhomogeneous surface distorts the spectral characteristics of the main pulse, which cannot be used for the detection and identification of the plastids. In (Figure 14c) the Reference signal spectrum is shown. As we can see, there are no minima in (Figure 14c), corresponding to absorption from environment, during the main pulse.

**Figure 15:**
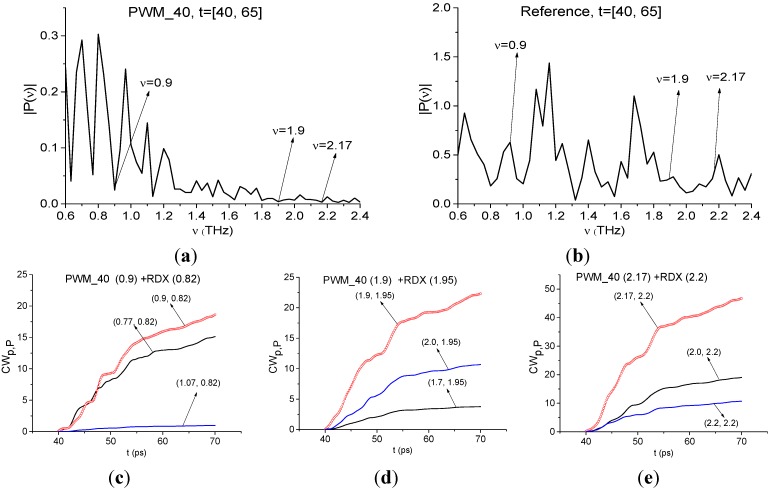
Fourier spectra of the first sub-pulse for the PWM_40 signal (**a**) and Reference signal (**b**) in the frequency range ν = [0.6, 2.4] THz, integral characteristics *CW_p,P_*(*t_n_*) calculated with the frequencies ν = 0.9 THz (**c**); 1.9 THz (**d**); 2.17 THz (**e**).

Below we show the possibility of plastids identification using a short time interval, containing the first sub-pulse *S*_1_(*t*) of the reflected THz signal, by means of integral correlation criteria Equations (5) and (6). With this aim, in Figure 15 the first sub-pulse spectrum for the PWM_40 signal (Figure 15a) and Reference (Figure 15b) are shown in the frequency range ν = [0.6, 2.4] THz.

We see that in Figure 15a there are minima at frequencies ν = 0.9, 1.9, 2.17 THz, that are close to the well-known absorption frequencies of the transmitted RDX signal ν = 0.82, 1.95, 2.19 THz [10] correspondingly. The difference between these frequencies may be caused by the frequency resolution Δν = 0.04 THz of our analysis, because of the short time interval *T* = 25 ps. In Figure 15b we see maxima of the Reference signal spectrum at these frequencies, which implies the absence of water vapor absorption. To find RDX in the PWM_40 signal we use the transmitted THz RDX_Air signal (see Figure 4) as the standard signal and spectral lines dynamics of this signal at frequencies ν = 0.82, 1.95, 2.2 THz. In Figure 15c,d the integral characteristic *CW_p,P_*(*t_n_*) is depicted detecting the frequencies ν = 0.9 THz (Figure 15c), 1.9 THz (Figure 15d), 2.17 THz (Figure 15e). In all cases (Figure 15c–e) the curves for the corresponding frequency pairs lie above others in the frequency range ν = [0.77, 1.07] THz (Figure 15c), [1.7, 2.0] THz (Figure 15d), [2.0, 2.2] THz (Figure 15e). Therefore, RDX is found in the PWM_40 signal corresponding to the pellet with rough surface. The same result ocurrs for the PWM_80 and PWM_120 signals.

**Figure 16:**
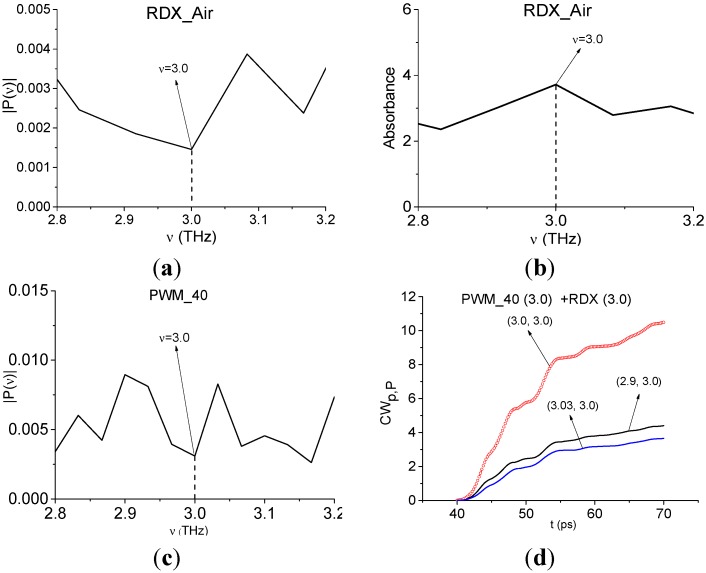
Fourier spectrum (**a**) and absorbance (**b**) of the RDX_Air signal, Fourier spectrum (**c**) of the first sub-pulse of the PWM_40 signal in the frequency range ν = [2.8,3.2] THz; integral characteristic *CW_p,P_*(*t_n_*) detecting the frequency ν = 3.0 THz (**d**).

Papers [6,7] reported about another absorption frequency for RDX: ν ≈ 3.0 THz. Figure 16 shows the Fourier spectrum (Figure 16a) and absorbance (Figure 16b) of the RDX_Air signal (Figure 16a) and the Fourier spectrum of the first sub-pulse PWM_40 (Figure 16c) in the frequency range ν = [2.8, 3.2] THz. One can see good agreement between the minimum (a), (c) and maximum (b) at the frequency ν = 3.0 THz. Therefore, we can use it for the identification of RDX. In Figure 16d the characteristic *CW_p,P_*(*t_n_*) calculated for the pair ν = (3.0, 3.0) THz, lies above others in the frequency range ν = (2.9, 3.03) THz. Thus, we see pronounced integral correlation of spectral dynamics of reflected PWM_40 and transmitted RDX_Air signals for various frequency pairs ν = (0.9, 0.82), (1.9, 1.95), (2.17, 2.2), (3.0, 3.0) THz in the time interval, containing the first sub-pulse of the PWM_40 signal. It means that RDX features are present in the PWM_40 signal.

**Figure 17:**
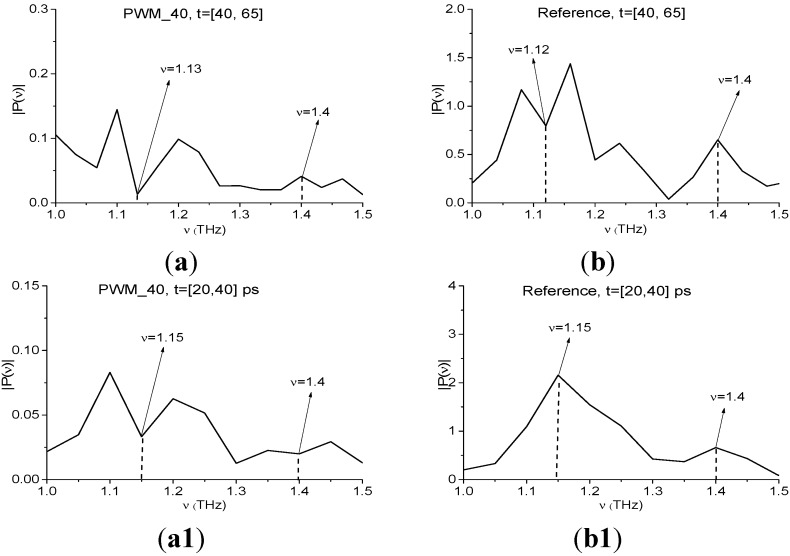

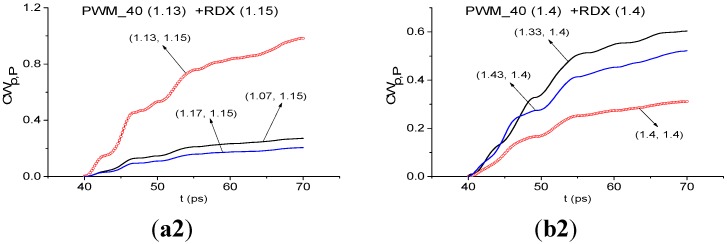
Fourier spectra of the PWM_40 signal (**a**) and Reference signal (**b**) in the time interval *t* = [40, 65] ps; Fourier spectra of the PWM_40 signal (**a1**) and Reference signal (**b1**) in the time interval *t* = [20, 40] ps; integral characteristics *CW_p,P_*(*t_n_*) detecting the frequencies ν = 1.13 THz (**a2**); 1.4 THz (**b2**) for PWM_40 and RDX_Air signals.

We showed above that the Fourier spectrum minima of the RDX_Air signal at frequencies ν = 1.15, 1.4 THz do not correspond to the absorption frequencies of pure RDX. They are caused by air water vapor present during the measurement. In the Fourier spectrum of the first sub-pulse of the PWM_40 signal (Figure 17a) a minimum at frequency ν = 1.13 THz ocurrs; at frequency ν = 1.4 THz there is a maximum. At the same time, in the Reference signal spectrum (Figure 17b) one can see the minimum at ν = 1.12 THz, which is close to ν = 1.13 THz. It implies the presence of some absorption from the environment at this frequency, but there is a maximum in both spectra at frequency ν = 1.4 THz, so the environment is transparent at the frequency ν = 1.4 THz. Thus, as for Reference signal, absorption of the PWM_40 signal at frequency ν = 1.13 THz may be caused by the influence of the environment. To clarify the possible reason for this effect, we present in (Figure 17a1,b1) the Fourier spectra of the PWM_40 signal and Reference signal in the time interval *t* = [20, 40] ps, which lies between the main pulse and the first sub-pulse of the signal PWM_40. In the spectrum of Reference signal (Figure 17b1) there is no minima at ν = 1.13 THz and ν = 1.4 THz, which implies the transparency of the environment. At the same time we see the absorption in the spectrum of PWM_40 signal at frequency ν = 1.15 THz, which may be caused by the influence of the molecules of water preserved on the rough surface of the sample and their complicated emission (we discussed this effect in the Introduction). Comparing spectra (Figure 17a) and (Figure 17a1) we see that the spectrum minimum at frequency ν = 1.15 THz in the time interval *t* = [20, 40] ps is preserved in the corresponding spectrum in the next time interval [40, 65] ps. The small difference between the values of the minima is caused by differences in spectral resolution, so they are Δν = 0.05 THz for the time interval *t* = [20, 40] ps and Δν = 0.04 THz for the time interval *t* = [40, 65] ps.

Figure 17 shows the characteristic *CW_p,P_*(*t_n_*) integral for the pair ν = (1.13, 1.15) THz (Figure 17a2) lying above other characteristics in the frequency range ν = [1.07, 1.17] THz; the integral characteristic for the pair ν = (1.4, 1.4) THz (Figure 17b2) lies below others in the range ν = [1.07, 1.17] THz. Detection of the frequency ν = 1.13 THz in (Figure 17a2) may be caused by the influence of the environment and energy absorption by water molecules on the sample surface. Figure 17b2 confirms that frequency ν = 1.4 THz is the false absorption frequency of RDX_Air.

**Figure 18:**
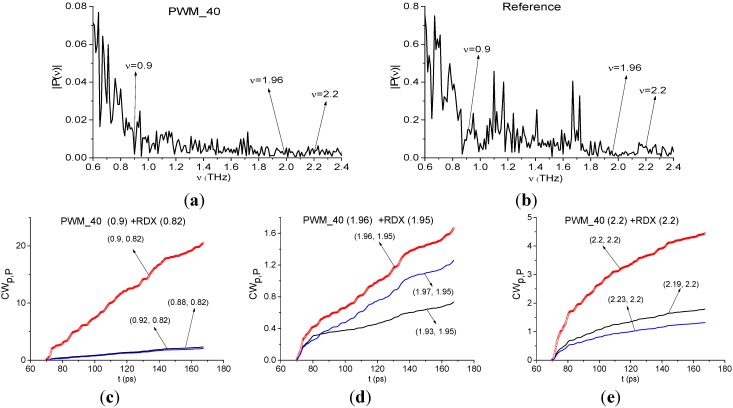
Fourier spectra of the PWM_40 signal (**a**) and Reference (**b**), calculated in the time interval t = [70, 170] ps, integral characteristics *CW_p,P_*(*t_n_*) detecting the frequencies ν = 0.9 THz (**c**); 1.96 THz (**d**); 2.17 THz (**e**).

Our next step is to show that one can identify plastic explosives even in the remote time interval *t* = [70, 170] ps, which doesn’t contain the first sub-pulse. Figure 18 shows the Fourier spectra of the PWM_40 signal (Figure 18a) and Reference signal (Figure 18b) calculated in this time interval. In Figure 18a one can see minima at frequencies ν = 0.9, 1.96, 2.2 THz. In Figure 18b minima, corresponding to the absorption frequencies of the environment, are absent. As above, we use the spectral lines dynamics of the RDX_Air signal at frequencies ν = 0.82, 1.95, 2.2 THz as standard ones.

In Figure 18c–e the integral characteristics *CW_p,P_*(*t_n_*) allow us to detect frequencies ν = 0.9 THz (Figure 18c), 1.96 THz (Figure 18d), 2.2 THz (Figure 18e) in the frequency ranges ν = [0.88, 0.92] THz (Figure 18c), [1.93, 1.97] THz (Figure 18d), [2.19, 2.23] THz (Figure 18e). It should be stressed that the frequency detection ranges were decreased in comparison with the previous case for the first sub-pulse.

**Figure 19:**
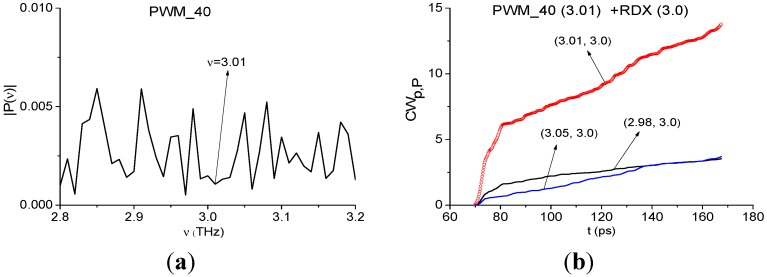
Fourier spectrum of the PWM_40 signal calculated in the time interval *t* = [70, 170] ps (**a**); integral characteristics *CW_p,P_*(*t_n_*) detecting the frequency ν = 3.01 THz (**b**).

The minimum at frequency ν = 3.01 THz is also present in the spectrum of the remote part of the PWM_40 signal (Figure 19a). As above, we use it for the identification. We see very clearly in (Figure 19b) the characteristic *CW_p,P_*(*t_n_*) for the pair ν = (3.01, 3.0) THz lies above others in the frequency range ν = [2.98, 3.05] THz.

**Figure 20:**
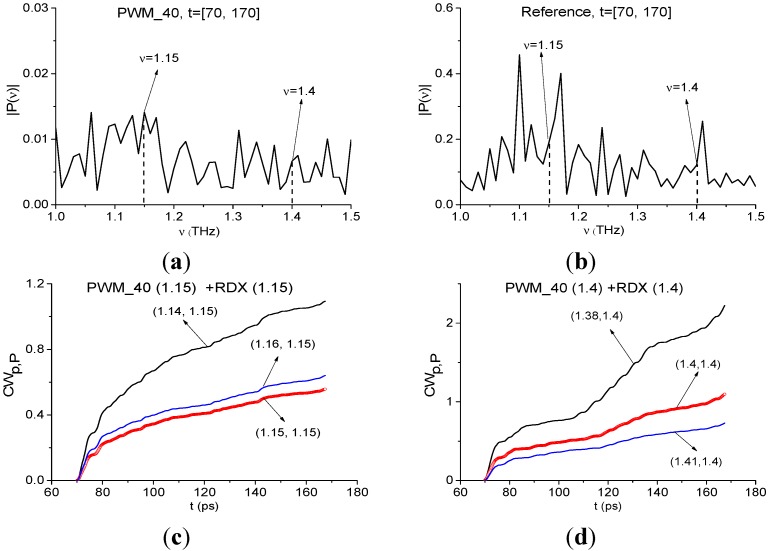
Fourier spectra of the remote part of the signal PWM_40 (**a**); Reference (**b**); integral characteristics *CW_p,P_*(*t_n_*) detecting the frequencies ν = 1.15 THz (**c**); ν =1.4 THz (**d**).

Figure 20 also shows the Fourier spectra of the remote part of the PWM_40 signal and Reference signal for *t* = [70, 170] ps. Unlike the previous case of identification using the first sub-pulse, the frequencies ν = 1.15, 1.4 THz do not correspond to the spectrum minima (Figure 20a), and there is no high absorption in the spectrum of Reference signal at ν = 1.15, 1.4 THz (Figure 20b). Integral characteristics *CW_p,P_*(*t_n_*) depicted in (Figure 20c,d), show that the frequencies ν = 1.15 THz and ν = 1.4 THz are not detected in the remote interval *t* = [70, 170] ps. It implies the absence of environmental absorptions in the time interval *t* = [70, 170] ps not containing the main pulse.

In the remote time interval *t* = [70, 170] ps we also observe a small shift of the absorption frequencies that we choose for the identification of the explosives, in comparison with the absorption frequencies of the standard RDX_Air signal: ν = 0.95, 1.95 THz are shifted to ν = 0.9, 1.96 THz for the PWM_40 signal. We believe that it may be caused by influence of the rough surface and spectral resolution Δν = 0.01 THz in the time interval under consideration.

Therefore, the integral correlation criteria Equations (5) and (6) allow us to find RDX in the PWM_40 sample with a rough surface both in the short time interval, containing the first sub-pulse, and in the remote time interval, which does not contain the first sub-pulse. The surface and the environment influences are manifested in the appearance of the THz radiation absorption at the frequency ν = 1.13 THz in the short time interval containing the first sub-pulse. We stress that RDX was detected in the PWM_80 and PWM_120 signals in the same way.

#### 4.2.2. Concave Surface

**Figure 21:**
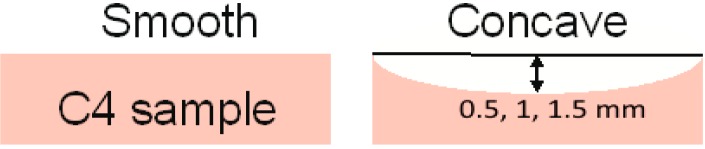
PWM C4 pellet with concave surface [32].

The next investigated case is the explosive PWM_C4 in pellets with concave surfaces with a curvature radius of 0.5, 1.0 or 1.5 mm (Figure 21). As above, the weight of all pellets with the PWM C4 is 600 mg. We name below the signals from the pellets as signals PWM_0.5, PWM_1.0 and PWM_1.5, for brevity. The signal structure is the same as in the previous case (see Figure 13).

**Figure 22:**
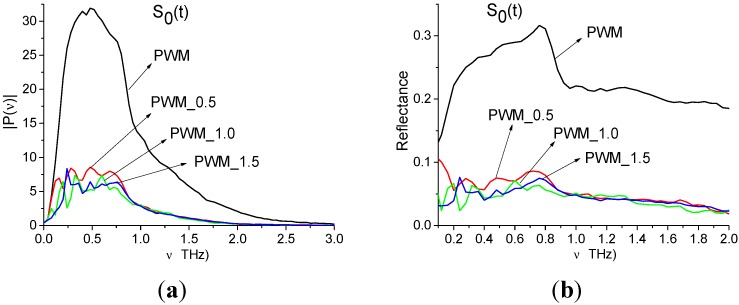
Fourier spectra (**a**) and reflectance (**b**) of the main pulses *S*_0_(*t*) of the PWM_0.5, PWM_1.0, PWM_1.5 and PWM signals [28].

In Figure 22 the Fourier spectra (Figure 22a) and reflectance (Figure 22b) of the main pulse *S*_0_(*t*) of the PWM_0.5, PWM_1.0, PWM_1.5 signals and smooth PWM are presented. Note that the spectral amplitudes in (Figure 22a) and the reflectance in (Figure 22b) of PWM_0.5, 1.0, 1.5 signals are much less than the spectral amplitude and reflectance of the smooth PWM signal, and the shape of spectra and reflectance of concave PWM signals in Figure 22a,b differs from that of the smooth PWM signal. Therefore, as in the case of the rough PWM_40, PWM_80 and PWM_120 signals, a concave surface also distorts the spectral properties of the main pulses of the PWM_0.5, PWM_1.0, PWM_1.5 signals and they cannot be used for identification.

As above, we study the spectral properties of the PWM_1.5 signal in two time intervals. The duration of the first interval is T = 25 ps and it contains only the first sub-pulse in 40 < *t* < 65 ps. The second time interval duration is T = 100 ps (70 < *t* < 170 ps) and it does not contain the first sub-pulse. In Figure 22 the Fourier spectrum (Figure 22a) of the first sub-pulse of the reflected THz PWM_1.5 signal and Reference signal spectrum (Figure 22b) are depicted in the frequency range ν = [0.6, 2.4] THz. The first sub-pulse spectrum contains minima at frequencies ν = 0.88, 1.92, 2.2 THz close to the true absorption frequencies of the transmitted RDX_Air signal at ν = 0.82, 1.95, 2.2 THz, correspondingly. Note that in (Figure 22a) there is another minimum, close to ν = 0.82 THz, at the frequency ν = 0.78 THz with almost the same value of spectral amplitude |*P*(ν)|, as the spectral amplitude for the minimum at ν = 0.88 THz. We choose both minima at frequencies ν = 0.78, 0.88 THz, close to ν = 0.82 THz, and will calculate integral characteristics *CW_p,P_*(*t_n_*) for both of them. Obviously in the Reference signal spectrum the minima at frequencies ν = 0.88, 1.92, 2.2 THz are absent, so environmental absorbance (for example, water vapor) is absent too.

**Figure 23:**
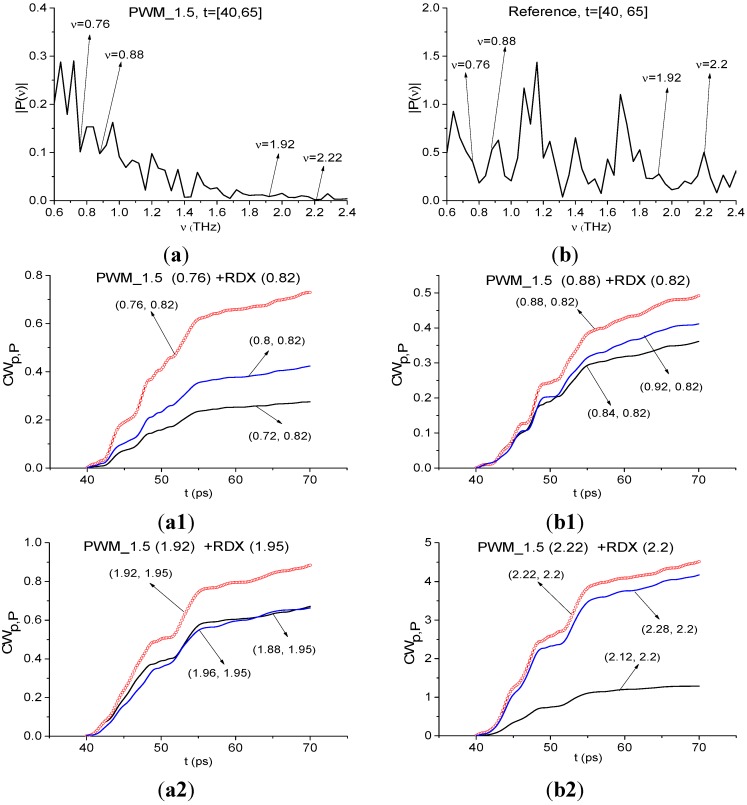
Fourier spectrum of the first sub-pulse of the PWM_1.5 signal (**a**) and Reference signal spectrum (**b**) in the frequency range ν = [0.6, 2.4] THz; integral characteristics *CW_p,P_*(*t_n_*) detecting the frequencies ν = 0.76 THz (**a1**); 0.88 THz (**b1**); 0.92 THz (**a2**); 2.22 THz (**b2**).

The spectral line dynamics of the transmitted RDX_Air signal at ν = 0.82, 1.95, 2.2 THz is used as the standard spectral line dynamics. In Figure 23a1,b1 the integral characteristics *CW_p,P_*(*t_n_*) are presented detecting the frequencies ν = 0.76 THz (Figure 23a1), 0.88 THz (Figure 23b1), 1.92 THz (Figure 23a2), 2.2 THz (Figure 23b2). The corresponding frequency ranges are ν = [0.72, 0.8] THz (Figure 23a1), [0.84, 0.92] THz (Figure 23b1), [1.88, 1.96] THz (Figure 23a2) and [2.12, 2.28] THz (Figure 23b2).

**Figure 24:**
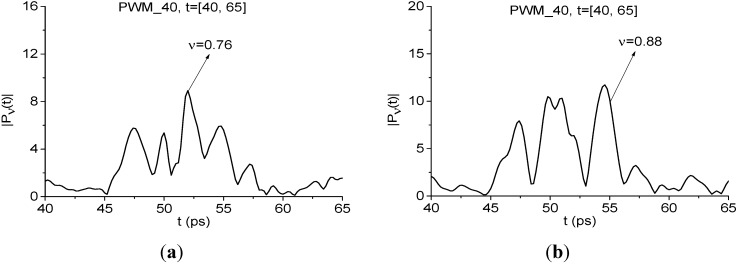
Dynamics of spectral lines of the PWM_1.5 signal at frequencies ν = 0.76 THz (**a**); ν = 0.88 THz (**b**).

It is interesting to note that both frequencies ν = 0.76, 0.88 THz demonstrate high integral correlation of the corresponding spectral dynamics with the dynamics of the standard RDX_Air signal at ν = 0.82 THz. The corresponding dynamics of the spectral lines of the PWM_40 signal are shown in Figure 24a,b.

The first reason for this lies in that the relaxation time at frequencies ν = 0.76 THz and 0.88 THz is the same. Nevertheless, the spectral line shape at the frequency ν = 0.88 THz is much closer to that for the RDX_Air signal at the frequency ν = 0.82 THz. Therefore, comparing the spectral lines dynamics of two signals allow us to identify the substance and gives one more possibility for this.

**Figure 25:**
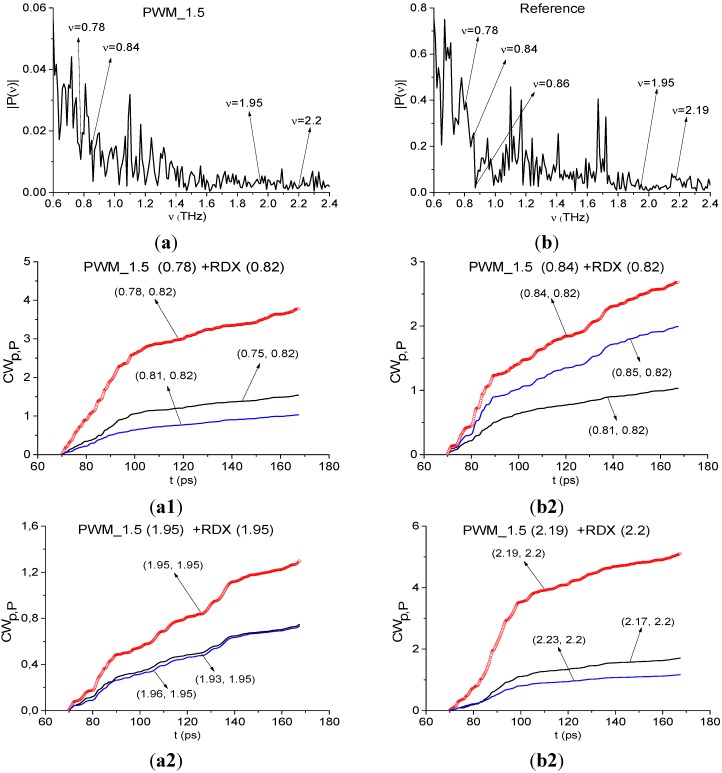
Fourier spectrum of the remote part of the PWM_1.5 signal (**a**) and Reference signal spectrum (**b**) in the frequency range ν = [0.6, 2.4] THz; integral characteristics *CW_p,P_*(*t_n_*) detecting the frequencies ν = 0.78 THz (**a1**); 0.84 THz (**b1**); 1.95 THz (**a2**); 2.19 THz (**b2**).

Figure 25 presents the Fourier spectrum of the PWM_1.5 signal (Figure 25a) and Reference signal spectrum (Figure 25b) calculated in the remote time interval *t* = [70, 170] ps. In Figure 25a, there are two minima at frequencies ν = 0.78, 0.84 THz, which are close to characteristic absorption frequency of RDX ν = 0.82 THz, and minima at ν = 1.95, 2.19 THz. In Figure 25b one can see the corresponding maxima of the Reference signal spectrum. The THz radiation absorption from the environment is absent at these frequencies. The deep minimum at the frequency ν = 0.86 THz in Figure 25a was out of our choice because there is an intensive absorption at this frequency in the Reference signal Figure 25b caused by the environment.

The integral characteristics *CW_p,P_*(*t_n_*) detect the frequencies ν = 0.78 THz (a1), 0.84 THz (b1), 1.95 THz (Figure 25a2), 2.22 THz (Figure 25b2). The frequency detection ranges were decreased in comparison with the case of plastic explosive identification using the first sub-pulse. The same situation ocurrs for the PWM_40 signal.

In the remote time interval we also found two frequencies ν = 0.78, 0.84 THz in the PWM_1.5 signal spectrum with a high integral correlation of the corresponding spectral dynamics with the spectral dynamics of the standard RDX_Air signal at ν = 0.82 THz. We believe that the reason is the same, as in the time interval containing the first-sub-pulse only—relaxation time at frequencies ν = 0.76 THz and ν = 0.84 Hz are close. We cannot exclude the influence of the surface shape on the appearance of these two frequencies. In this case, it is necessary to compare the spectral intensity evolution for assessment of the RDX presence in the signal under consideration. The small differences in absorption frequencies used for identification may be caused by spectral resolution differences. Its value is Δν = 0.04 THz for the time interval, containing the first sub-pulse of the signal PWM_1.5, and Δν = 0.01 THz for the remote time interval.

**Figure 26:**
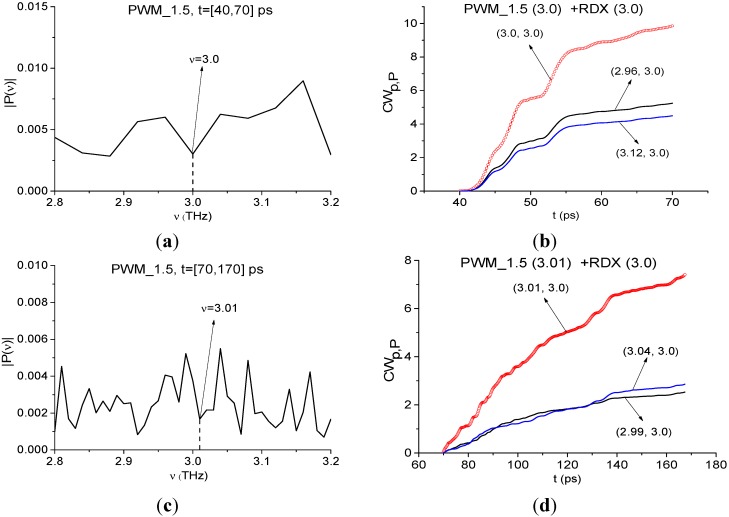
Fourier spectra of the first sub-pulse (**a**) and the remote part (**c**) of the PWM_1.5 signal in the frequency range ν = [2.8, 3.2] THz; integral characteristics *CW_p,P_*(*t_n_*) detecting the frequencies ν = 3.0 THz (**b**); 3.01 THz (**d**) in the time intervals *t* = [40,70] ps (**b**) and *t* = [70,170] ps (**d**).

In Figure 26 one can see the spectrum minimum at frequency ν = 3.0 THz for the first sub-pulse of the PWM_1.5 signal (Figure 26a) and at ν = 3.01 THz for the remote part of this signal (Figure 26c). In Figure 26b,d the characteristic *CW_p,P_*(*t_n_*) detects frequencies ν = 3.0 THz (Figure 26b) and 3.01 THz (Figure 26d) in the frequency ranges ν = [2.96, 3.12] THz and [2.99, 3.04] THz, correspondingly.

We see the high integral correlation of the spectral line dynamics for the concave PWM_1.5 and transmitted RDX_Air signals in the time interval *t* = [40, 70] ps, containing the first sub-pulse only, and in the remote time interval *t* = [70, 170] ps. The corresponding detected frequencies are: ν = 0.76, 0.88, 1.92, 2.2, 3.0 THz for the first time interval and ν = 0.78, 0.84, 1.95, 2.19, 3.01 THz for the second one. The environmental influence as well as the complicated shape of the sample surface are expressed in the presence of two pairs of frequencies with high integral correlation of the corresponding spectral dynamics with the standard spectral lines dynamics at frequency ν = 0.82 THz. At the same time, high frequencies ν =2.2 (2.19) THz, 3.0 (3.01) THz show less dependence on the surface shape and the environment. Therefore, they are more useful for reliable substance identification.

#### 4.2.3. Limitations of the Proposed Method

**Figure 27:**
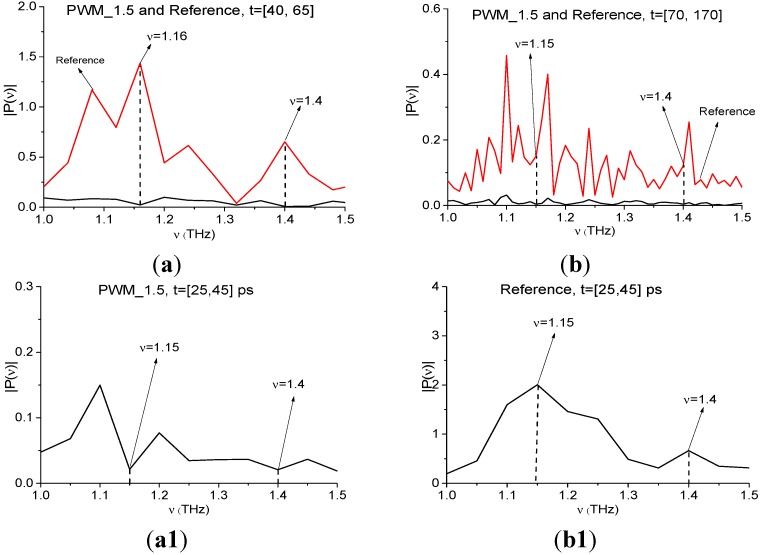
Fourier spectra of the PWM_1.5 signal and Reference signal, calculated in the time intervals t = [40, 65] (**a**) and t = [70, 170] (**b**); spectrum of the signal PWM_1.5 (**a1**) and Reference signal (**b1**) for t = [25, 45] ps.

Figure 27 shows the Fourier spectra of the PWM_1.5 signal and Reference signal, calculated in the time intervals *t* = [40, 65] (Figure 27a) and *t* = [70, 170] (Figure 27b). Frequencies ν = 1.16, 1.4 THz are the spectrum minima of the PWM_1.5 signal in the time interval *t* = [40, 65] ps (Figure 27a), containing the first sub-pulse; and ν =1.15, 1.4 THz are the spectrum minima of the remote part of the signal *t* = [70, 170] ps (Figure 27b). At the same time, we do not see minima at these frequencies in the corresponding Reference signal spectrum (Figure 27a), which means the absence of THz radiation absorption from the environment during the first sub-pulse.

We have seen such a situation in Section 4.2.1 for the first sub-pulse spectrum of the PWM_40 signal at frequency ν = 1.15 THz. As in that Section, in figures (a1,b1) we present Fourier spectra of the PWM_1.5 signal and Reference signal, calculated in the time interval, lying between the main pulse and the first sub-pulse. For the signal PWM_1.5 it is *t* = [25, 45] ps. The Reference signal spectrum (b1) contains maxima at frequencies ν = 1.15, 1.4 THz, that indicate the environment is transparent for the THz signals at these frequencies. At the same time, we see an absorption in the PWM_1.5 signal spectrum at ν = 1.15, 1.4 THz, and it is not caused by the environment, so molecules of water preserved on the sample surface cause absorptions at these frequencies. Both minima are present in the time interval t = [40, 65] ps. Small differences of the minima values are caused by spectral resolution differences, which is Δν = 0.05 THz for the time interval *t* = [25, 45] ps and Δν = 0.04 THz for the time interval *t* = [40, 65] ps.

However, in the remote time interval absorption from the environment is present, because in Figure 27b one can see the minima of the Reference signal spectrum at frequencies ν = 1.14, 1.39 THz. Therefore, the THz radiation absorption of the PWM_1.5 signal in the remote part *t* = [70, 170] is caused by the environment.

**Figure 28:**
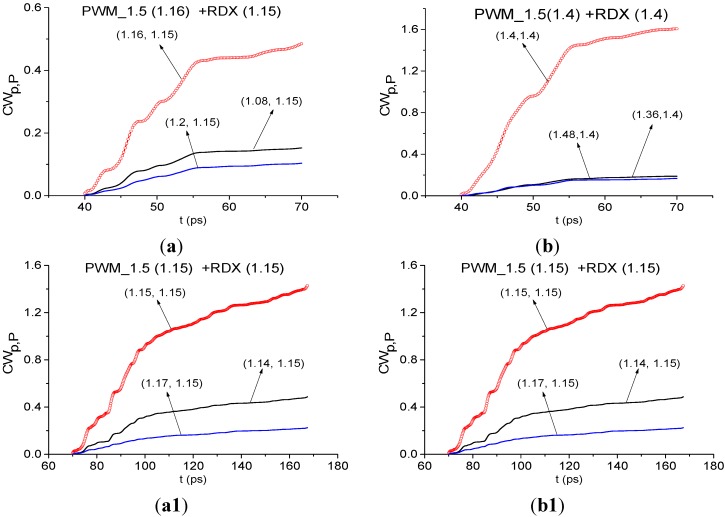
Integral characteristics detecting the frequencies ν = 1.16 THz (**a**); 1.4 THz (**b**) in the time interval *t* = [40, 65] ps; ν = 1.15 THz (**a1**); 1.4 THz (**b1**) in the time interval *t* = [70, 170] ps.

In Figure 28 the integral characteristic *CW_p,P_*(*t_n_*) detects the frequency ν = 1.16 THz in the interval *t* = [40, 65] ps (Figure 28a), ν =1.15 THz—in the remote interval *t* = [70, 170] ps (Figure 28a1). The frequency ν = 1.4 THz is detected in both time intervals (Figure 28b,b1). Note that the reasons for the detection in both cases are different—the influence of the water molecules on the concave surface during the first sub-pulse and the influence of the environment in the remote part of the signal.

Thus, despite the fact that we do not use a reference signal for calculating the integral characteristics, in some cases for reliable identification it is necessary to analyze the reference signal spectrum in order to exclude the influence of water vapor and environment.

Let us note several more potential limitations of the method. Some difficulties with the application of the integral correlation criteria for the identification of substances may be associated with the emergence of substances, which have dynamics of spectral intensities similar to the spectral line dynamics of studied substance. However, it is possible to overcome this disadvantage by extending the number of characteristic frequencies that can be used for identification.

To date, the proposed method has been successfully applied for the identification of substances at distances of up to 3.5 m under real conditions. Considering that using this method one can obtain useful information from noisy parts of the signal, we expect that this distance may be increased to 10 m. However, with further increase of the distance between the object and the receiver, due to attenuation of the coherent signal the identification of substances may become difficult.

## 5. Conclusions

We showed the essential restriction of the standard THz TDS method for detection and identification, which is based on comparing the spectra of substances, by using paper layers as an example of substance under analysis. In our opinion, the THz TDS method is insufficient for the reliable identification of substances under real conditions (at long distance and high relative humidity) and may incorrectly interpret the information obtained. At the same time, the proposed integral criteria and method of spectral dynamics analysis allow us to detect the absence of dangerous substances and to detect paper in the sample.

The integral criteria were applied in the reflection mode for the identification of the explosive PWM C4 with an inhomogeneous surface using the time intervals which do not contain the main pulse of the reflected THz pulse with a few cycles. The transmitted RDX_Air signal was used as a standard one. We showed that it is possible to detect and identify this explosive. For reliable identification of the substance, it is necessary to use for computer processing only those parts of the reflected THz signal, which do not contain the main pulse. The analysis of the reflected signal must be performed over the long-term time interval (more than 100 ps). Previously, nobody has been able to identify with high probability PWM C4 with an inhomogeneous surface by analyzing its Fourier spectrum and reflectance.

We demonstrated that the inhomogeneous surface and environment strongly affect the identification if we use for this the low range frequencies, which are close to the RDX absorption frequency ν = 0.82 THz. It should be stressed that the high frequencies close to ν = 2.2, 3.0 THz show independence from the influence of the surface shape and the environment, therefore, they are preferable for reliable identification.

Under real conditions, identification of a substance from the noisy THz signal can occur. The amplitude of the useful signal can be compared with the amplitude of noise, which is caused by the influence of the atmosphere and the equipment. The THz signal measured under laboratory conditions also contains noise, caused by the equipment. However, the noisy signal always contains the response of the medium, and our goal was to find the spectral fingerprints of the substance in this signal.

Note that we have conducted a series of experiments with other neutral substances in addition to paper layers (with chocolate, cookies, thick paper bags) under real conditions—and showed that the integral correlation criteria allow us to detect the absence of explosives and drugs in neutral substances as well as to find sugar in chocolate and cookies [30,33]. These experiments confirm the reproducibility of the results presented in the article.

Thus, the proposed method, which can be named a “terahertz nose”, is a promising and competitive method for the effective detection and identification of dangerous substances, including explosives with complicated surfaces, in comparison with the THz TDS method, based on comparison of the substance spectra. The method can be used with success to solve security problems, and for the problem of non-destructive testing, as well as for quality control in the pharmaceutical industry.

## References

[1] Kemp M.C., Taday P.E., Cole B.E., Cluff J.A., Fitzgerald A.J., Tribe W.R. (2003). Security applications of terahertz technology. Proc. SPIE.

[2] Tribe W.R., Newnham D.A., Taday P.E., Kemp M.C. (2004). Hidden object detection: Security applications of terahertz technology. Proc. SPIE.

[3] Zimdars D., White J.S. (2004). Terahertz reflection imaging for package and personnel inspection. Proc. SPIE.

[4] Federici J.F., Schulkin B., Huang F., Gary D., Barat R., Oliveira F., Zimdars D. (2005). THz imaging and sensing for security applications—Explosives, weapons and drugs. Semicond. Sci. Technol..

[5] Startsev M.A., Elezzabi A.Y. (2013). Terahertz frequency continuous-wave spectroscopy and imaging of explosive substances. ISRN Opt..

[6] Shen Y.C., Lo T., Taday P.E., Cole B.E., Tribe W.R., Kemp M.C. (2005). Detection and identification of explosives using terahertz pulsed spectroscopic imaging. Appl. Phys. Lett..

[7] Leahy-Hoppa M.R., Fitch M.J., Zheng X., Hayden L.M., Osiander R. (2007). Wideband terahertz spectroscopy of explosives. Chem. Phys. Lett..

[8] Davies A.G., Burnett A.D., Fan W., Linfield E.H., Cunningham J.E. (2008). Terahertz spectroscopy of explosives and drugs. Mater. Today..

[9] Liu H.B., Chen Y., Bastiaans G.J., Zhang X.C. (2006). Detection and identification of explosive RDX by THz diffuse reflection. Opt. Express.

[10] Chen J., Chen Y., Zhao H., Bastiaans G.J., Zhang X.C. (2007). Absorption coefficients of selected explosives and related compounds in the range of 0.1–2.8 THz. Opt. Express.

[11] Palka N. (2011). THz reflection spectroscopy of explosives measured by time domain spectroscopy. Acta Phys. Pol. A.

[12] Palka N. (2014). Identification of concealed materials, including explosives, by terahertz reflection spectroscopy. Opt. Eng..

[13] Xiong W., Shen J. (2010). Fingerprint extraction from interference destruction terahertz spectrum. Opt. Express.

[14] Ortolani M., Lee J.S., Schade U., Hübers H.W. (2008). Surface roughness effects on the terahertz reflectance of pure explosive materials. Appl. Phys. Lett..

[15] Xu J., Plaxco K.W., Allen S.J. (2006). Absorption spectra of liquid water and aqueous buffers between 0.3 and 3.72 THz. J. Chem. Phys..

[16] Trofimov V.A., Varentsova S.A. (2007). New method for analysis of temporal dynamics of medium spectrum under the action of terahertz pulse. Proc. SPIE.

[17] Trofimov V.A., Varentsova S.A. (2009). About efficiency of reconstruction of materials using spectrum dynamics of medium response under the action of THz radiation. Proc. SPIE.

[18] Trofimov V.A., Varentsova S.A., Chen J. (2010). Identification of explosive using the spectrum dynamics of reflected THz and GHz radiation. Proc. SPIE.

[19] Trofimov V.A., Varentsova S.A. (2010). 2D THz signature for substance identification. Pros. SPIE.

[20] Trofimov V.A., Varentsova S.A., Krotkus A., Molis G. (2010). Identification of substance in complicated mixture of simulants under the action of THz radiation on the base of SDA (Spectral Dynamics Analysis) method. Proc. SPIE.

[21] Trofimov V.A., Varentsova S.A., Shen J., Zhang C., Zhou Q., Shi Y. (2011). 2D signature for identification of drugs. Proc. SPIE.

[22] Trofimov V.A., Varentsova S.A., Palka N., Szustakowski M., Trzcinski T. (2011). The method of the spectral dynamics analysis of reflected signal for problem of identification of substance. Proc. SPIE.

[23] Trofimov V.A., Varentsova S.A., Palka N., Szustakowski M., Trzcinski T. (2011). Efficiency of the detection of explosive using the spectral dynamics analysis of reflected signal. Proc. SPIE.

[24] Trofimov V.A., Varentsova S.A., Szustakowski M., Palka N. (2012). Efficiency of the detection and identification of ceramic explosive using the reflected THz signal. Proc. SPIE.

[25] Trofimov V.A., Varentsova S.A., Szustakowski M., Palka N. (2012). Detection and identification of compound explosive using the SDA method of the reflected THz signal. Proc. SPIE.

[26] Trofimov V.A., Peskov N.V., Kirillov D.A. (2012). Efficiency of using correlation function for estimation of probability of substance detection on the base of THz spectral dynamics. Proc. SPIE.

[27] Trofimov V.A., Varentsova S.A., Palka N., Szustakowski M., Trzcinski T., Lan S., Liu H.Y. (2011). An influence of the absolute phase of THz pulse on linear and nonlinear medium response. Proc. SPIE.

[28] Trofimov V.A., Varentsova S.A., Szustakowski M., Palka N. (2013). Influence of surface of explosive on its detection and identification using the SDA method for analysis of the reflected THz signal. Proc. SPIE.

[29] Trofimov V.A., Varentsova S.A. (2013). Effective criteria for the identification of substance using the spectral lines dynamics of reflected THz signal. Proc. SPIE.

[30] Trofimov V.A., Varentsova S.A., Trofimov V.V., Tikhomirov V.V. (2014). Peculiarities of the detection and identification of substance at long distance. Proc. SPIE.

[31] Trofimov V.A., Varentsova S.A., Trofimov V.V. (2014). Transmission of THz pulse with a few circles through opaque samples placed at long distance (4–6 m). Proc. SPIE.

[32] Trofimov V.A., Varentsova S.A. (2014). Real-time criteria based on spectral dynamics of medium response for the detection and identification of substance using THz signal. Proc. SPIE.

[33] Trofimov V.A., Varentsova S.A., Trofimov V.V. (2014). Possibility of the detection and identification of substance at long distance at using broad THz pulse. Proc. SPIE.

[34] Kemp M.C., Baker C., Gregory I. (2006). NATO Security through Science Series.

[35] Singh S. (2007). Sensors—An effective approach for the detection of explosives. J. Hazard. Mater..

[36] Buryakov I.A., Buryakov T.I., Matsaev V.T. (2014). Optical chemical sensors for the detection of explosives and associated substances. J. Anal. Chem..

[37] Onodera T., Toko K. (2014). Towards an electronic dog nose: Surface plasmon resonance immunosensor for security and safety. Sensors.

[38] Li T., Patz A., Mouchliadis L., Yan J., Lograsso T.A., Perakis I.E., Wang J. (2013). Femtosecond switching of magnetism via strongly correlated spin-charge quantum excitations. Nature.

[39] Luo L., Chatzakis I., Patz A., Wang J. (2015). Ultrafast terahertz probes of interacting dark excitons in chirality-specific semiconducting single-walled carbon nanotubes. Phys. Rev. Lett..

